# Health in All Policies Implementation in Quebec Suggests Great Promise for U.S.

**DOI:** 10.5334/aogh.5058

**Published:** 2026-04-17

**Authors:** Keshia M. Pollack Porter, Stefanie Carignan, Pierre-Gerlier Forest

**Affiliations:** 1 Johns Hopkins Bloomberg School of Public Health, Health In All Policies Initiative, Baltimore, MD, USA; 2 Institut national de santé publique du Quebec, Montreal, QC, Canada

In the U.S., high spending on healthcare in recent decades has not yielded gains in health outcomes commensurate with expenditures. The U.S. spends nearly twice as much on healthcare as the average OECD country, but health outcomes measured by several indicators are among the lowest [1]. States in the U.S. spend a significant proportion of their budget on Medicaid, which provides health insurance to eligible low‑income adults, children, older adults, and people with disabilities, among others [2]. Federal changes to Medicaid in the One Big Beautiful Bill Act, which was signed into law in July 2025, are projected to reduce coverage and increase states’ costs [3]. These policy changes could impact health outcomes and health equity, as people may be less able to afford health insurance or access healthcare.

We argue that states can mitigate sweeping changes to federal policy and make their constituents healthier by implementing Health in All Policies (HiAP). Working “upstream” and intentionally integrating health considerations into non‑health sectors is an effective way to improve health outcomes and prevent illness and injury, which can reduce reliance on costly emergency department visits for medical care (i.e., “downstream” impacts) over time. HiAP is one way to achieve optimal well‑being and equity by systematically addressing the health implications of decisions in a variety of sectors that affect health, such as social services like childcare, as well as sectors like transportation and housing.

HiAP has been implemented in many countries, which presents an opportunity for learning and application to the U.S. using a global learning for health equity framework [4]. One component of this framework is considering the cultural, historical, political, and economic contexts where the global approaches were developed. In doing this, we examine Quebec, Canada’s largest province by area, which has embraced HiAP or the notion of healthy public policies for decades, for lessons on how to effectively adapt this strategy to the U.S. We conclude that HiAP has the potential, when fully implemented, to advance health equity in the U.S.

## Quebec’s Health in All Policies Approach

Despite its distinct language and traditions, Quebec is very much a part of North America. Its institutions, landscape, and economy, as well as essential aspects of its culture, have been shaped by its integration into Canada and its adoption of many American values and ways of life. It is not the poorest province in the Canadian federation, but it is not on the same footing as industrial Ontario or petrol‑rich Alberta. In effect, its *per capita* GDP makes it look more like Alabama or Arkansas than New York or Massachusetts, the wealthiest states in the U.S. [5]. Yet Quebec has achieved some of the best health outcomes observed on our continent, with numbers close to countries with long social democratic traditions.

Life expectancy at birth in Quebec (82.65 years) [6] exceeds that of Hawaii (80 years), the U.S.’s best‑performing state in this metric [7]. Those results were not achieved by chance [8]. One explanation is the decision, in the early 1970s, to keep health and social public programs under the authority of the same minister and consequently, to encourage the two sectors to maintain a constant dialogue on needs, resources, and services. The bold and fully integrated health and wellness policies of the 1990s, the first of their kind in Canada, are the fruit of this dynamic [9]. The other reason is the firm commitment of the province of Quebec, a few years later, to the principles of HiAP [10]. Under the leadership of ministers of different political affiliations, the province adopted a law that imposed the health impact appraisal of every new piece of legislation (2001) [11], a comprehensive provincial public health program (2003, 2008, and 2015), and, in 2016, one of the first governmental policies ever implemented claiming HiAP as its inspiration and road map [12].

Quebec’s HiAP strategy targets the root causes of noncommunicable diseases. Goals include promoting early childhood development best practices, building communities that increase their residents’ quality of life, addressing emotional and mental well‑being, and incorporating disease prevention principles into health and social services [13]. These efforts are ongoing, with a new government‑wide prevention strategy enacted in the fall of 2025, soon to be followed by a revised public health program.

Quebec governments of that period were formed mostly by right of center, pro‑business political parties. No doubt they would *not* have followed the same course if the case for an integrated approach to healthy policies was less obvious, not only in terms of both outcomes and healthcare costs, but also for living conditions and overall prosperity. What they agreed to support was a steady commitment to coordination among all parts of government—at the level of deputy ministers, where decisions can be made or changed—and the intellectual discipline that comes with the recourse to evidence before action. The notion that this approach could produce far‑reaching benefits without a heavy investment of financial and human resources was a defining aspect of its success with the province’s leadership.

## Implications for the U.S.

Although the U.S. has not seen health outcomes commensurate with its spending on healthcare, results from Quebec illuminate a potential path forward that would cost less in the long run. To continue the comparison between Quebec and Alabama, whose health outcomes are dramatically different despite similar GDPs, annual healthcare expenditures in Quebec are lower at approximately US$6,407 per person, compared to US$9,821 in Alabama, where total life expectancy is 9 years shorter (as of 2022) [7, 14, 15]. Further explaining Quebec’s achievements in population health, a U.S.‑based study found that greater spending on social services relative to healthcare bore out significant benefits for health outcomes [15]. Based on this, we ask what can the U.S. learn from Quebec’s experience? We highlight three points for consideration.

First, leadership must be committed to bridging silos and working across sectors to find innovative and effective solutions to advance health. These collaborations can be facilitated by multisector workgroups or advisory councils that bring health and non‑health sectors together. In Quebec, HiAP has been implemented to emphasize collaboration, where the health sector is considered a convenor rather than a leader. This approach is critical because it counters criticism that HiAP centers health in a way that raises concerns of health imperialism, where the “health sector presumptively and arrogantly takes leadership and assumes that health is everyone’s main priority” [16]. In the U.S., efforts to bring sectors together, where representatives from the health sector embrace humility, learning, listening, and engagement with other sectors can help build trust to identify shared goals and foster intersectoral and multisector approaches [17].

Second, integration of health and wellness in policy decisions requires intention. HiAP uses a variety of tools and Quebec’s HiAP approach is backed by legislation that requires a health impact assessment (HIA) be conducted on policies within and outside of the health sector [16, 18]. HIA “is a combination of procedures, methods and tools by which a policy, program or project may be judged as to its potential effects on the health of a population, and the distribution of those effects within the population” [19]. In the U.S., most HIAs are not mandated, but are conducted voluntarily by academic institutions, governmental, or nongovernmental organizations [20]. However, this still creates an opportunity to voluntarily use HIAs or related tools that apply a health lens to policy proposals [21]. To advance health equity, HIAs or similar analyses could be conducted on policies that address the underlying drivers of health inequities, including uneven distribution of power, sexism, or racism.

Third, and perhaps most importantly, given the current political climate, advancing HiAP does not have to be partisan. As experienced in Quebec, there was a commitment to promoting HiAP under conservative, moderate, and progressive ministers [22]. This demonstrates that advancing policies that promote rather than harm health is an approach that resonates with diverse political perspectives, especially when the benefits for the economy and society, as well as overall health and well‑being are highlighted. HiAP is a framework with great potential to benefit the health and well‑being of all communities, everywhere.

## Conclusions

Advancing health equity in the U.S. requires innovative approaches to policymaking that address the drivers of poor health. Lessons from other countries with contexts similar to the U.S., such as Canada, show the importance of working across sectors, intentionally considering health, and promoting a narrative that HiAP can deliver on priorities across the political spectrum. However, cultural norms may pose a challenge to implementation. The success of HiAP frameworks is contingent on understanding and prioritizing collective well‑being, and can conflict with the U.S. “bootstrapping” mentality.

A review of the impacts of HiAP in Quebec suggests great promise for improving health and health equity in the U.S. HiAP’s potential to reduce inequities in noncommunicable diseases, morbidity, and mortality rates in the U.S. could be realized through strengthening intersectoral and multisectoral collaboration to bring together health and non‑health sectors; and intentionally using tools like HIA to bring a health lens to decisions outside of the health and healthcare sectors. HiAP is an effective nonpartisan approach to policymaking with many health, social, and economic benefits.
